# Supplementary material for: The Role of Nutrient Supplements in Female Infertility: An Umbrella Review and Hierarchical Evidence Synthesis
Source: Nutrients. 2024 Dec 27;17(1):57. doi: 10.3390/nu17010057 (PMC11722770; doi:10.3390/nu17010057)
Supplement: Supplementary file 1 [file nutrients-17-00057-s001.zip › nutrients-3340184-supplementary.pdf]

Table S1. Sample search strategy for umbrella review examining nutrient supplements for female fertility

	Population (P)	Intervention (I)	Type of Study	Restrictions
**Keyword search words used (' OR ' terms)**	1.exp infertility/<br>2. exp/IVF (In-Vitro fertilization)<br>3.exp/ART (Assisted reproductive technology)<br>4.exp/unexplained infertility	Nutrient supplements* OR vitamin* OR mineral* OR dietary supplement* OR nutrient* OR food supplement* OR nutritional supplement* OR health supplement* OR multivitamin* OR omega 3 OR fish oil* OR alpha*lipoic acid OR alpha*linolenic acid* acetylcysteine OR cysteine OR probiotic* OR tryptophan OR tocopherol OR alphatocopherol OR carotene OR retinol OR thiamine OR riboflavin OR niacin OR niacinamide OR nicotinic acid OR pantothenic OR pyridox* OR biotin OR methylfolate OR 5-MTH*OR levomefolic acid OR folate OR folinic acid OR folic acid OR inositol OR cyanocobalamin OR methylcobalamin OR cobalamin OR ascorbic acid OR cholecalciferol OR iron OR ferrous OR tocopherols OR trace element OR calcium OR phosphorus OR magnesium OR potassium OR manganese OR zinc OR selenium OR boron OR chromium OR lycopene OR isoflav* OR flavonoid* OR bioflavonoid* OR micronutrient* OR carnitine acid OR alpha linoleic acid OR eicosapentaenoic OR docosahexaenoic	Umbrella review*<br>Meta-review*OR<br>Meta-analy*OR<br>Metaanaly*OR<br>meta reg* OR<br>metareg* OR<br>meta-reg* OR<br>systematic review*	Human<br>Female<br>Last 5 years<br>English language

Table S2. Critical domains for AMSTAR 2 [1]

Critical domains for AMSTAR 2	Definition
Item 2	Establishment of review methods prior to conducting the review and reporting any significant deviations from the protocol
Item 4	Adequate literature search in at least two bibliographic databases and search strategy available on request
Item 7	Justification for excluding individual studies
Item 9	Risk of bias from individual studies being included in the review
Item 11	Appropriateness of meta-analytical methods
Item 13	Consideration of risk of bias when interpreting the results of the review
Item 15	Assessment of presence and likely impact of publication bias

(1) Shea BJ, Reeves BC, Wells G, Thuku M, Hamel C, Moran J, et al. AMSTAR 2: a critical appraisal tool for systematic reviews that include randomised or non-randomised studies of healthcare interventions, or both. *BMJ*. 2017;358:j4008. https://doi.org/10.1136/bmj.j4008

Table S3. List of excluded studies

Lead Author	Published Year	Title	Reason for exclusion
Abdoli, S.	2020	Adverse quality of lifestyle and risk of infertility: A systematic review study	Non-English studies
Abodi, M.	2022	Omega-3 fatty acids dietary intake for oocyte quality in women undergoing assisted reproductive techniques: A systematic review	Wrong intervention
Alesi, S.	2022	Nutritional Supplements and Complementary Therapies in Polycystic Ovary Syndrome	Wrong study design
Andraos, S.	2018	The Impact of Nutritional Interventions in Pregnant Women on DNA Methylation Patterns of the Offspring: A Systematic Review	Wrong setting
anonymous	2017	Erratum: Prevalence of Vitamin B-12 insufficiency during pregnancy and its effect on offspring birth weight: A systematic review and meta-analysis (American Journal of Clinical Nutrition (2016) 103 (1232-51)	Wrong setting
anonymous	2018	Evidence for prenatal vitamin D supplementation too weak to make recommendations	Wrong setting
Antunes, M. R.	2022	Effect of the anti-inflammatory diet in the control of oxidative stress and its relation with quality of life and gestational success in patients with endometriosis	Conference abstract
Arhin, S. K.	2017	Effect of micronutrient supplementation on IVF outcomes: a systematic review of the literature	Wrong patient population
Athe, R.	2020	Meta-analysis approach on iron fortification and its effect on pregnancy and its outcome through randomized, controlled trials	Wrong study design
Bala, R.	2021	Hyperhomocysteinemia and low vitamin B12 are associated with the risk of early pregnancy loss: A clinical study and meta-analyses	Wrong study design
Bayar, E.	2020	Impact of polyunsaturated fatty acid supplementation on assisted reproductive technology (ART) outcomes: a systematic review	A more recent study was included
Bernstein L.R.	2021	Editorial: Causes of Oocyte Aneuploidy and Infertility in Advanced Maternal Age and Emerging Therapeutic Approaches	Wrong setting
Berti, C.	2018	Multiple-micronutrient supplementation: Evidence from large-scale prenatal programmes on coverage, compliance and impact	Non-English studies
Bi, W. G.	2018	Association between Vitamin D supplementation during pregnancy and offspring growth, morbidity, and mortality: A systematic review and meta-analysis	Wrong patient population
Bi, W. G.	2018	Vitamin D supplementation during pregnancy and offspring mortality and morbidity: A systematic review	Wrong patient population
Bialy, L.	2020	Vitamin D supplementation to improve pregnancy and perinatal outcomes: an overview of 42 systematic reviews	Wrong patient population
Biswas, K.	2021	Selenium supplementation in pregnancy-maternal and newborn outcomes: A systematic review	Wrong patient population
Bosdou, J. K.	2019	Vitamin D and obesity: Two interacting players in the field of infertility	Wrong study design
Brannon, P. M.	2017	Iron supplementation during pregnancy and infancy: Uncertainties and implications for research and policy	Wrong setting
Buch, S.	2021	The influence of omega-3 fatty acids on female fertility in assisted reproductive technology	Wrong study design
Burchakov, D. I.	2017	Omega-3 long-chain polyunsaturated fatty acids and preeclampsia: Trials say "No," but is it the final word?	Wrong setting
Cairncross, Z. F.	2019	Measurement of Preconception Health Knowledge: A Systematic Review	Wrong study design

Candido, Aline C.	2019	Insufficient iodine intake in pregnant women in different regions of the world: a systematic review	Wrong patient population
Cardoso, B. R.	2023	Nut consumption and fertility: a systematic review and meta-analysis	Wrong intervention
Cavoretto P.I.	2022	Time to implement vitamin D assessment and supplementation into routine obstetric practice?	Wrong study design
Chakhtoura, M.	2018	Vitamin D in the Middle East and North Africa	Wrong patient population
Cheng, Z.	2022	Evaluation of the association between maternal folic acid supplementation and the risk of congenital heart disease: a systematic review and meta-analysis	Wrong outcomes
Chowdhury, M. H.	2020	Effect of supplementary omega-3 fatty acids on pregnant women with complications and pregnancy outcomes: review from literature	Wrong patient population
Christiansen, C. H.	2022	Multivitamin use and risk of preeclampsia: A systematic review and meta-analysis	Wrong patient population
Chu, J.	2018	Vitamin D and assisted reproductive treatment outcome: A systematic review and meta-analysis	Wrong study design
Chu, X.	2022	Probiotics for preventing gestational diabetes mellitus in overweight or obese pregnant women: A systematic review and meta-analysis	Wrong outcomes
Ciebiera, M.	2021	Nutrition in gynecological diseases: Current perspectives	Wrong study design
Corbett, G. A.	2021	Probiotic therapy in couples with infertility: A systematic review	Wrong study design
Corbett, G. A.	2022	Nutritional interventions to ameliorate the effect of endocrine disruptors on human reproductive health: A semi-structured review from FIGO	Non English studies
Cozzolino, M.	2020	How vitamin D level influences in vitro fertilization outcomes: results of a systematic review and meta-analysis	Wrong study design
Cozzolino, M.	2020	Therapy with probiotics and synbiotics for polycystic ovarian syndrome: a systematic review and meta-analysis	Wrong outcomes
Curtis, E. M..	2018	Maternal Vitamin D supplementation during pregnancy	Wrong patient population
Devi, N.	2019	Effect of N-acetylcysteine supplementation on women undergoing assisted reproductive techniques: A systematic review and meta-analysis	Wrong setting
Di Renzo, L.	2022	Immunonutrients involved in the regulation of the inflammatory and oxidative processes: implication for gamete competence	Wrong study design
Di Tucci, C.	2021	The role of alpha lipoic acid in female and male infertility: a systematic review	Wrong patient population
Diamanti-Kandarakis, E	2017	Nutrition as a mediator of oxidative stress in metabolic and reproductive disorders in women	Wrong setting
Dolmans, M. M.	2021	Conservative management of uterine fibroid-related heavy menstrual bleeding and infertility: Time for a deeper mechanistic understanding and an individualized approach	Wrong study design
Donovan, S.	2020	Omega-3 fatty acids from Supplements Consumed before and during Pregnancy and Lactation and Developmental Milestones, Including Neurocognitive Development, in the Child: A Systematic Review	Wrong outcomes
Elnashar, A.	2019	Antioxidants for female infertility: Review of systematic reviews	Conference abstract
Facchinetti, F.	2020	Breakthroughs in the Use of Inositols for Assisted Reproductive Treatment (ART)	Wrong study design
Falsaperla, R.	2017	Pyridoxine supplementation during pregnancy, lactation and the first months of life: A review of the literature	Wrong patient population
Filipowicz, D.	2021	The rationale for selenium supplementation in patients with autoimmune thyroiditis, according to the current state of knowledge	Wrong patient population

Firouzabadi, F. D.	2022	The effects of omega-3 polyunsaturated fatty acids supplementation in pregnancy, lactation, and infancy: An umbrella review of meta-analyses of randomized trials	Wrong patient population
Fite, M. B.	2021	Compliance with Iron and Folic Acid Supplementation (IFAS) and associated factors among pregnant women in Sub- Saharan Africa: A systematic review and metaanalysis	Wrong patient population
Florou, P.	2020	Does coenzyme Q10 supplementation improve fertility outcomes in women undergoing assisted reproductive technology procedures? A systematic review and meta-analysis of randomized-controlled trials	A more recent study was included
Fogacci, F.	2020	Safety Evaluation of alpha-Lipoic Acid Supplementation: A Systematic Review and Meta-Analysis of Randomized Placebo-Controlled Clinical Studies	Wrong patient population
Gatti, C. R.	2021	Unsaturated Fatty Acid Intake During Periconception and Incidence of Achieving Pregnancy: A Systematic Review and Meta-Analysis	No risk of bias analysis
Goto, E.	2019	Effectiveness of prenatal lipid-based nutrient supplementation to improve birth outcomes: A Meta-analysis	Wrong patient population
Habib, N.	2022	Impact of lifestyle and diet on endometriosis: a fresh look to a busy corner	Wrong study design
Hantoshzadeh, S.	2017	The effect of vitamin D on fertility	Wrong study design;
Harding K.B.	2017	Iodine supplementation for women during the preconception, pregnancy and postpartum period	Wrong outcomes
Hibberd, R.	2018	Re: Inositol treatment of anovulation in women with polycystic ovary syndrome: a meta-analysis of randomised trials	Wrong study design
Hofmeyr, G. J.	2019	Calcium supplementation commencing before or early in pregnancy, for preventing hypertensive disorders of pregnancy	Wrong study design
Holt-Kentwell, A.	2022	Evaluating interventions and adjuncts to optimize pregnancy outcomes in subfertile women: an overview review	A more recent study was included
Iliuta, F.	2022	Women's vitamin D levels and IVF results: a systematic review of the literature and meta-analysis, considering three categories of vitamin status (replete, insufficient and deficient)	Non-English studies
Kalaitzopoulos, D. R.	2022	Effects of vitamin D supplementation in endometriosis: a systematic review	Overlapping studies
Kalra, B. S.	2019	Supplemental Antioxidants: A Hype in Disease Prevention	Wrong setting
Kirsneris, P.	2018	Relationship between vitamin D deficiency and fertility damage	Wrong setting
Kuma, M. N.	2022	Effect of Nutrition Interventions Before and/or During Early Pregnancy on Low Birth Weight in Sub-Saharan Africa: A Systematic Review and Meta-Analysis	Wrong patient population
Lagan A.S.	2017	Evidence-based and patient-oriented inositol treatment in polycystic ovary syndrome: Changing the perspective of the disease	Wrong setting
Ley, D.	2021	Iodine supplementation: is there a need?	Wrong setting
Lima, L. G.	2022	Relation between Selenium and Female Fertility: A Systematic Review	Non-English studies
Lindenberg S.	2022	Are we close enough to understanding and diagnosing polycystic ovarian syndome?	Wrong setting
Llaneza, P.	2018	Comparison of the effect of two combinations of myo-inositol and D-chiro-inositol in women with polycystic ovary syndrome who undergo ICSI	Wrong study design
Lopez-Moreno, A.	2020	Probiotics dietary supplementation for modulating endocrine and fertility microbiota dysbiosis	Wrong patient population
Lopez-Moreno, A.	2021	Vaginal probiotics for reproductive health and related dysbiosis: Systematic review and meta-analysis	Wrong patient population

Lucchetta, R. C.	2022	Deficiencia e insuficiencia de vitamina D em mulheres na idade reprodutiva: Uma revisao sistematica e meta-analise, Deficiency and Insufficiency of Vitamin D in Women of Childbearing Age: A Systematic Review and Meta-analysis	Non-English studies
Lv, Q.	2021	Advances in Research on the Toxicological Effects of Selenium	Wrong study design
Maleki, V.	2019	Potential roles of carnitine in patients with polycystic ovary syndrome: a systematic review	Wrong outcomes
Manouchehri, A.	2023	Polycystic ovaries and herbal remedies: A systematic review	Wrong outcomes
Mascarenhas, M.	2021	Concordance between systematic reviews of randomized controlled trials in assisted reproduction: An overview	Wrong outcomes
Mejlhede, M.	2020	Melatonin - a new fertility wonder drug? A systematic review on the impact of melatonin on fertility	Wrong study design
Mendoza, N.	2017	Inositol supplementation in women with polycystic ovary syndrome undergoing intracytoplasmic sperm injection: a systematic review and meta-analysis of randomized controlled trials	A more recent study was included
Menichini, D.	2022	Nutraceuticals and polycystic ovary syndrome: a systematic review of the literature	No risk of bias analysis
Mojaverrostami, S.	2019	The role of melatonin in polycystic ovary syndrome: A review	Animal study
Moridi, I.	2020	The association between vitamin d and anti-mullerian hormone: A systematic review and meta-analysis	No risk of bias analysis
Morshed-Behbahani, B.	2023	Effect of and the association between vitamin D and outcomes of assisted reproductive techniques among infertile men and women: protocol for an overview of systematic reviews and meta-analysis	Wrong study design
Murugesu, S.	2017	Does the use of calcium ionophore during artificial oocyte activation demonstrate an effect on pregnancy rate? A meta-analysis	Wrong study design
Najafi, M. N.	2018	Phytoestrogens and the polycystic ovary syndrome: A systematic review of clinical evidence and laboratory findings	Wrong intervention
Nasiadek, M.	2020	The role of zinc in selected female reproductive system disorders	Wrong setting
Norman, R. J.	2023	Management of subfertility in women with PCOS: expectations, problems and solutions	Conference abstract
Oso, C.	2020	Sunny side up? The role of vitamin d in follicular development in women of advanced maternal age undergoing intrauterine insemination	Conference abstract
Paffoni, A.	2022	Folate Levels and Pregnancy Rate in Women Undergoing Assisted Reproductive Techniques: a Systematic Review and Meta-analysis	Wrong study design
Papageorghiou, A.T.	2020	From evidence to implementation	Wrong setting
Pundir, J.	2019	Overview of systematic reviews of non-pharmacological interventions in women with polycystic ovary syndrome	Overlapping studies
Pundir. J.	2018	Authors' reply re: Inositol treatment of anovulation in women with polycystic ovary syndrome: a meta-analysis of randomised trials	Wrong study design
Pundir, J.	2018	Inositol treatment of anovulation in women with polycystic ovary syndrome: a meta-analysis of randomised trials	Published earlier than 2017
Safinejad, H.	2017	Assessing the effect of vitamin D and calcium on the treatment of infertility in women with polycystic ovary syndrome (PCOD): A review article	Conference abstract
Scutiero, G..	2017	Oxidative Stress and Endometriosis: A Systematic Review of the Literature	No risk of bias analysis
Seifert-Klauss, V.	2017	Vitamin D and live birth rate after IVF	Wrong setting
Shen, C.	2019	The relationship between Vitamin D and IVF: A systematic review and meta-analysis	Wrong study design

Shin, H. W.	2021	Comparative efficacy and safety of intravenous ferric carboxymaltose and iron sucrose for iron deficiency anemia in obstetric and gynecologic patients: A systematic review and meta-analysis	Wrong outcomes
Showell, M. G.	2018	Inositol for subfertile women with polycystic ovary syndrome	Overlapping studies
Simpson, S.	2023	Vitamin D and infertility	Wrong setting
Snoek, K. M.	2020	How does bariatric surgery influence maternal periconception health? A systematic review	Wrong intervention
Souza, R. C. V. E.	2021	The Influence of Nutrients Intake during Pregnancy on Baby's Birth Weight: A Systematic Review	Wrong patient population
Sudfeld, C. R.	2019	New evidence should inform WHO guidelines on multiple micronutrient supplementation in pregnancy	Wrong patient population
Taghavi, S. A.	2021	Pharmacological and non-pharmacological strategies for obese women with subfertility	Wrong intervention
Tamblyn, J. A.	2022	Vitamin D and miscarriage: a systematic review and meta-analysis	Wrong patient population
Tan, X.	2022	Magnesium supplementation for glycemic status in women with gestational diabetes: a systematic review and meta-analysis	Wrong patient population
Taylor, B. L.	2017	Effect of probiotics on metabolic outcomes in pregnant women with gestational diabetes: A systematic review and meta-analysis of randomized controlled trials	Wrong patient population
Tenorio, M. B.	2018	Oral antioxidant therapy for prevention and treatment of preeclampsia: Meta-analysis of randomized controlled trials	Wrong patient population
Toolan, M.	2022	A systematic review and narrative synthesis of antenatal interventions to improve maternal and neonatal health in Nepal	Wrong patient population
Tuenter, A.	2019	Folate, vitamin B12, and homocysteine in smoking-exposed pregnant women: A systematic review	Wrong patient population
Vitagliano, A.	2021	Dietary supplements for female infertility: A critical review of their composition	Wrong study design;
Whynott, R. M.	2017	The Effect of Uterine Fibroids on Infertility: A Systematic Review	No risk of bias analysis
Wu, J. H.	2021	The effect of oral vitamin E supplementation on infertile women: A systematic review and meta-analysis	A more recent study was included
Yadav, K.	2020	Comparison of different doses of daily iron supplementation for anemia prophylaxis in pregnancy: A systematic review	Wrong patient population
Yang, Y.	2021	The effect of prepregnancy body mass index on maternal micronutrient status: a meta-analysis	Wrong study design
Zace, D.	2022	A comprehensive assessment of preconception health needs and interventions regarding women of childbearing age: a systematic review	Wrong study design
Zhang, C.	2017	Dietary iron intake, iron status, and gestational diabetes	Wrong patient population
Zhang, H.	2019	The efficacy of myo-inositol supplementation to prevent gestational diabetes onset: a meta-analysis of randomized controlled trials	Wrong patient population
Zhang, Y.	2020	Adjuvant treatment strategies in ovarian stimulation for poor responders undergoing IVF: a systematic review and network meta-analysis	A more recent study was included
Zhao, J.	2018	Whether vitamin D was associated with clinical outcome after IVF/ICSI: A systematic review and meta-analysis	Wrong study design
Zheng, X.	2017	Inositol supplement improves clinical pregnancy rate in infertile women undergoing ovulation induction for ICSI or IVF-ET	A more recent study was included
Zhou, L.	2021	Probiotics and synbiotics show clinical efficacy in treating gestational diabetes mellitus: A meta-analysis	Wrong patient population

Zhou, S.	2017	Vitamin D and risk of preterm birth: Up-to-date meta-analysis of randomized controlled trials and observational studies	Wrong patient population
Zhou, X.	2022	Effect of Vitamin D Supplementation on In Vitro Fertilization Outcomes: A Trial Sequential Meta-Analysis of 5 Randomized Controlled Trials	A more recent study was included
Zhu, F.	2023	TEAS, DHEA, CoQ10, and GH for poor ovarian response undergoing IVF-ET: a systematic review and network meta-analysis	Overlapping studies
